# Ant Abundance along a Productivity Gradient: Addressing Two Conflicting Hypotheses

**DOI:** 10.1371/journal.pone.0131314

**Published:** 2015-07-15

**Authors:** Udi Segev, Jaime Kigel, Yael Lubin, Katja Tielbörger

**Affiliations:** 1 Institute for Plant Sciences, Robert H. Smith Faculty of Agriculture, Food and Environment, The Hebrew University of Jerusalem, Rehovot, Israel; 2 Mitrani Department of Desert Ecology, Blaustein Institutes for Desert Research, Ben-Gurion University of the Negev, Midreshet Ben-Gurion, Israel; 3 Plant Ecology Department, University of Tübingen, Tübingen, Germany; Universidade de São Paulo, Faculdade de Filosofia Ciências e Letras de Ribeirão Preto, BRAZIL

## Abstract

The number of individuals within a population or community and their body size can be associated with changes in resource supply. While these relationships may provide a key to better understand the role of abiotic vs. biotic constraints in animal communities, little is known about the way size and abundance of organisms change along resource gradients. Here, we studied this interplay in ants, addressing two hypotheses with opposite predictions regarding variation in population densities along resource gradients- the ‘productivity hypothesis’ and the ‘productivity-based thinning hypothesis’. The hypotheses were tested in two functional groups of ground-dwelling ants that are directly primary consumers feeding on seeds: specialized seed-eaters and generalist species. We examined variations in colony density and foraging activity (a size measurement of the forager caste) in six ant assemblages along a steep productivity gradient in a semi-arid region, where precipitation and plant biomass vary 6-fold over a distance of 250km. An increase in the density or foraging activity of ant colonies along productivity gradients is also likely to affect competitive interactions among colonies, and consequently clinal changes in competition intensity were also examined. Ant foraging activity increased with productivity for both functional groups. However, colony density revealed opposing patterns: it increased with productivity for the specialized seed-eaters, but decreased for the generalist species. Competition intensity, evaluated by spatial partitioning of species at food baits and distribution of colonies, was uncorrelated with productivity in the specialized seed-eaters, but decreased with increasing productivity in the generalists. Our results provide support for two contrasting hypotheses regarding the effect of resource availability on the abundance of colonial organisms- the ‘productivity hypothesis’ for specialized seed-eaters and the ‘productivity-based thinning hypothesis’ for generalist species. These results also stress the importance of considering the role of functional groups in studies of community structure.

## Introduction

One of the major goals of ecology is to understand the processes that account for variation in community structure along geographical gradients [1,2]. Among these, patterns of variation in species abundance have been the focus of many broad-scale ecological studies. Many of these studies have shown that taxocene (i.e. taxonomically related set of species co-occurring in a given place) abundance could co-vary positively with resource availability along primary productivity gradients (lizards: [3], ants: [4], birds: [5]), thus supporting the premise that greater food availability can maintain greater population densities. This hypothesis was termed the ‘productivity hypothesis’ [4]. Nevertheless, other studies failed to find such evidence [6] or have even found a decrease in population densities along productivity gradients [7,8].

A lack of positive correlation between productivity and abundance suggests that this relationship can be affected by factors other than resource availability alone. For example, in many taxocenes, body size has been shown to influence the way species use and divide resources and therefore could also limit their relative abundances [9–12]. Moreover, mean body size of individuals could increase with increasing resources, as has been shown by several studies (mammals: [13], ants: [14], birds: [15]). Consequently, increased resource supply can support more individuals in a population or community but it can also lead to an increase in body size of organisms [10,16], in which case abundance might remain unchanged or even decrease. Thus, along resource gradients, energetic tradeoffs could affect size-abundance relationships due to density-dependent processes, such as resource competition among organisms in the community [10,12]. We therefore suggest that along productivity gradients, taxocene abundance might not be constrained only by resource availability but also by changes in mean body size of individuals which could affect density-dependent processes, such as competition for limited resources. We refer to this hypothesis as the ‘productivity-based thinning hypothesis’. The different predictions of these two hypotheses call for in-depth studies of clines of abundance and size along resource gradients. However, while these relationships can provide a key to understanding the underlying processes that affect community structure, our knowledge on the way size and abundance of animals vary along resource gradients is still limited.

We studied the relationship between abundance and productivity in ground-dwelling ants (Formicidae) with regards to the above two hypotheses, in an attempt to explain the patterns of variation in their abundance along a productivity gradient in a semi-arid region. Ants are ideally suited to address this question as they are a common taxonomic group in most terrestrial ecosystems [17] and are sensitive to environmental changes [4,18,19]. In ants, which are eusocial insects, abundance is measured as *colony density* (number of colonies area^-1^) [4]. Accordingly, as suggested in the productivity hypothesis, if colony density is constrained by resource availability alone, it is predicted to increase with increasing resources. In contrast, the productivity-based thinning hypothesis suggests that colony density might be constrained not only by resource availability, but also by changes in mean body size of individuals (i.e. the size of ant colonies), which could limit colony density due to resource depletion or interference competition among colonies. In ants, the size of a colony comprises both the mass of individual workers and the number of workers [14]. As a size measurement in this study we used *foraging activity* per colony (number of foragers colony^-1^ time^-1^), a size estimate of the forager caste.

Previous studies have shown that an increase in foraging activity and resource intake of ant colonies could be associated with increases in primary productivity, particularly in systems that are resource limited [20–24]. Increased foraging activity can in turn result in increased competition among neighboring colonies due to increased interference and aggressiveness, which may affect the monopolization of food sources and the success of colonies such that colonies with more workers are more aggressive and can better defend their space or foraging ranges [25–30]. This suggests that density dependent interactions, such as competition for shared resources among species with similar ecological requirements, could lead to lower densities in the more productive sites than would be expected according to site productivity. Accordingly, the productivity-based thinning hypothesis proposes that in the more productive sites, ant colonies could be better able to defend space or resources due to the predicted increase in foraging activity of colonies, which may result in a lower colony density in these sites. The predictions of this hypothesis could be directly linked to total colony size (number of workers per colony) or colony biomass (the product of worker number per colony and mean worker body mass), but also could be associated with foraging activity, because all energy gain per colony is a product of foraging [4,31,32]. In addition, competition between colonies occurs mainly via exploitation of food resources and behavioral interactions among foragers (e.g. [17,21,27,33]).

To summarize, the productivity hypothesis predicts that colony density is limited by the amount of available resources and will increase with increasing resources along a productivity gradient. Alternatively, the productivity-based thinning hypothesis predicts that colony density is limited by foraging activity as well as resource availability and therefore that colony density will decrease with increasing foraging activity of ant colonies in the assemblage along a productivity gradient.

As an increase in the density or foraging activity of ant colonies is likely to affect competitive interactions among colonies along productivity gradients, we examined several predictions regarding the way competition intensity varies in ant assemblages in response to changes in the availability of resources. For instance, if colony density increases with increased resources, as suggested in the productivity hypothesis, competition intensity might increase as well due to increased aggressiveness among the more densely aggregated colonies. However, competition also could be intense at lower levels of resource availability, due to the 'struggle' for the limited resources. Alternatively, if colony density decreases with increased resources, as suggested in the productivity-based thinning hypothesis, competition intensity might remain unchanged with an increase in resource availability due to the expected trade-off between colony density and foraging activity. Previous studies tested the way competition intensity varies along stress gradients in several communities, such as plants (e.g. the stress-gradient hypothesis, [34]) and ants [35–37]. In the latter, competition was found to be a major factor regulating assemblages in low-stress regions as defined by ambient temperature. However, there is still no solid conceptual foundation for determining how competition intensity varies in ant assemblages along productivity gradients at a macroecological scale. This is of particular importance in low productivity regions, where primary stressors for plants (i.e. water availability) and ants (i.e. food availability) are associated.

Here, we examined variation in colony density and foraging activity as well as variation in an estimate of competition intensity between ant colonies in six assemblages along a natural productivity gradient occurring over a relatively short geographic distance in a semi-arid region. This productivity gradient exhibits large variation in precipitation and biomass of the herbaceous vegetation (Table 1) nested within a single regional pool of ant species and a similar habitat type (e.g. [38]). Along this geographic gradient, productivity varies greatly while the temperature range remains fairly constant (Table 1). The lack of a temperature gradient is important here, since along macroecological gradients ant community abundance was found to be influenced mostly by variation in primary productivity and temperature [4]. In particular, temperature, together with precipitation, act as the main predictors of primary productivity [39], and consequently could affect the amount of available resources. Temperature could also limit the ability of foragers to access food resources [4], through effects on foraging activity and efficiency (e.g. [27,40]) as well as individual metabolic rates [41]. This study system provides an opportunity to study the effect of primary productivity independently from that of ambient temperature, where precipitation is the main driver of site productivity. In order to generalize our findings, we studied these interactions for two consecutive years in two functional groups of ground-dwelling ants that use similar resources but differ in their body sizes (S1 Fig) and their ecological requirements: generalist species *vs*. specialized seed eaters. In our study system primary productivity is composed mainly of annual plants, whose seeds are the main source of food for both generalist and specialist ant species. We predicted that the specialized seed eaters, which use seeds as a greater fraction of their diet, have larger and highly polymorphic foragers and are expected to be more efficient in foraging for seeds [27,40,42], would have higher rates of resource acquisition (e.g. [27,31]) and would therefore exhibit a higher rate of increase in foraging activity along the productivity gradient compared to the generalist species.

**Table 1:** Study sites and their characteristics.

Site	Location	Average annual precipitation (mm) (average ± SE)*	Average monthly temperature (°C)^†^	Plant biomass (g/m^2^/y) (average ± SE)^‡^	Dominant annual plant species
**Sede Boqer**	30°51' N / 034°47' E	94 ± 40 (150,69)	18.7 (12–25)	65 ± 21 (113,57)	*Stipa capensis*, *Erucaria microcarpa*
**Hatzerim**	31°15' N / 034°39' E	197 ± 60 (182,111)	20.8 (14–27)	149 ± 48 (290,110)	*Stipa capensis*, *Onobrychis squarrosa*
**Lahav**	31°23' N / 034°52' E	308 ± 100 (285,203)	19.6 (14–25)	198 ± 53 (326,103)	*Avena sterilis*, *Hordeum spontaneum*
**Amatzia**	31°32' N / 034°52' E	380 ± 140 (400,246)	19.6 (14–25)	195 ± 42 (253,114)	*Aegilops* sp., *Hordeum spontaneum*
**Karei Deshe**	32°55' N / 035°35' E	550 ± 145 (421,310)	22.5 (16–29)	244 ± 50 (280,149)	*Hordeum bulbosum*, *Avena sterilis*
**Ramot Menashe**	32°33' N / 035°06' E	662 ± 185 (599,503)	20.3 (16–25)	370 ± 76 (375,243)	*Hordeum spontaneum*, *Daucus carota*

* Average annual precipitation refers to the years 1978–2008 (Israel Meteorological Service archives). Rainfall amounts for the years 2007 and 2008 are in parentheses.

† Average monthly temperature refers to average monthly temperatures between the years 1998–2008 (IMS archives). Average monthly minimum and maximum temperatures between the years 1998–2008 are presented in parentheses.

‡ Plant biomass is the average of 12 0.6×0.6 m samples per site per year for the years 2007–2010. NPP amounts for the sampled years are in parentheses.

## Materials and Methods

### Study sites

The study was carried out in six sites located at low-elevation (at 135–470 m a.s.l.) along a south-north ecocline across mesic-Mediterranean and desert regions in Israel. These regions have a Mediterranean bi-seasonal climate, with a mild and rainy winter growth season (October-March), and a warm dry summer season. Precipitation in the study sites along the ecocline increases from 94 mm × year^-1^ in the Negev Desert up to 662 mm × year^-1^ in the Mediterranean region, within a ca. 250 km transect, while mean annual temperatures remain similar along the ecocline (Table 1). Minimum and maximum distances between the nearest sites are 37±18 km and 68±40 km, respectively.

Productivity, i.e. the rate at which energy flows through an ecosystem [43], can be assessed indirectly in several ways, such as precipitation, evapotranspiration and plant biomass [44]. Here we used mean annual precipitation (averaged for the years 1978–2008), which is commonly used as a surrogate of productivity in a variety of studies in this arid region (e.g. [40,43,45]). The precipitation gradient corresponded to an increase in plant biomass, which was assessed by measuring the dry weight of above-ground biomass of herbaceous plants at peak season (April) in 12 0.6×0.6 m quadrats per site per year, during four consecutive years (2007–2010). There was a significant correlation between mean annual precipitation for the years 1978–2008 and biomass of herbaceous plants (Spearman’s rank coefficient = 0.94, P = 0.005, n = 6 sites; Fig a in S2 Fig), as well as between mean annual precipitation and actual precipitation for each year (Spearman’s rank coefficient = 1.00, P < 0.001, n = 6 for the years 2007 or 2008). However, no correlation was found between annual precipitation and ambient temperature (Spearman’s rank coefficient = 0.10, P = 0.85, n = 6; Fig b in S2 Fig), or between plant biomass and ambient temperature (Spearman’s rank coefficient = 0.10, P = 0.85, n = 6). In order to reduce potential effects of habitat heterogeneity, the study sites were restricted to open habitats with herbaceous vegetation and high dominance of annual and hemicryptophytic species, and were located in Long-Term Ecological Research stations or in private rangelands, to minimize anthropogenic disturbance. Permission to access sites was given by private land owners and by the Blaustein Institutes for Desert Research, Ben-Gurion University; Hatzerim Airbase; Lehavim LTER station and Northern Research and Development, Galilee Technology Center. This study did not involve endangered or protected species.

### Ant colony density and foraging activity

Estimations of colony density and foraging activity were taken at different spatial scales for the generalist species (three 20 × 15 m plots per site) and the specialized seed eaters (one 100 × 100 m plot per site). We used these two spatial scales since the specialized seed eaters, with nest mounds extending up to 1–2 m in diameter ([46], per. obs.) and distances to nearest colonies between 7 m (Karei Deshe site) to 13 m (Sede Boqer site), were infrequently represented in the smaller plots. In addition, colony density measurements were also taken for the specialized seed eaters at the 20 × 15 m plots, together with the generalist species.

#### Generalist species

Workers of most of these ant species collect both plant material, such as seeds or fruits, and dead arthropods [46,47]. Species included in this group were generalist foragers, most of which belong to three functional groups according to both global [35,48] and regional [46,47] classifications: Generalized Myrmicinae (the genera *Crematogaster*, *Monomorium* and *Pheidole*), Opportunists (*Tetramorium*) and Dominant Dolichoderinae (*Tapinoma*) (S1 Table). *Camponotus* and *Cataglyphis* species were excluded from the analysis since they were poorly sampled by our methods due to their foraging behavior (i.e., single foragers) and because they are ecologically distinct from the other functional groups in that they do not feed on seeds or plant material (feeding mainly on honey-dew collectors and dead arthropods, respectively; [48]).

The foraging activity of the generalist species was monitored in each site at three randomly-chosen plots of 20 × 15 m located at least 100 m apart. The sampling was conducted in two consecutive years at the same plots, during August 2007 (mid- summer) and September 2008 (end of summer), when ant activity is high [38], but before the first rains and the foundation of new colonies by new foundresses. There were large differences in yearly amounts of precipitation between these two years; precipitation per site decreased by 20–50% along the gradient from the Mediterranean to the desert between 2007 and 2008 (Table 1).

Colony density and forager number per colony were evaluated by monitoring ant foraging activity in the plots during a 24-h cycle. Twenty food baits were placed 5 m apart in each plot at sunset. Each food bait consisted of a 0.2 × 0.2 m paper sheet covered with separate patches of honey (5 g) at one end and sesame and millet seeds (5 g) at the other. In 2007, the baits were sampled during the night (22:00) and the following morning (06:00) two and ten hours after bait placement, respectively. In 2008, the baits were sampled once during the night (22:00) and twice during the following morning (06:00, 09:00) due to an increase in the duration of ant activity. Due to bait destruction by jackals in 2007, one plot was excluded from each of three sites (Lahav, Karei Deshe and Ramot Menashe; Table 1). The food baits were observed for approximately two minutes, during which the number of individuals occupying the baits were counted per species. Workers were identified to species on-site and in cases of uncertainty were collected, stored in vials with 70% ethanol and identified in the laboratory using identification keys. Nomenclature followed the Hymenoptera Online Database (<http://hol.osu.edu>). Voucher specimens are found in the authors’ collection.

Colony density was estimated according to the number of active nests per plot. Active nests were evaluated and marked by tracking workers returning to their nests from the food-baits. Nest entrances of the same species that were less than 1 m apart were counted as a single nest entrance (e.g. [4]). Least-squares linear regression was used to test the relationship between mean number of colonies per plot averaged for the years 2007–2008 and mean annual precipitation. This analysis was tested separately for the generalist species and for both functional groups combined.

Forager number per colony was assessed as the number of workers counted along all foraging trails found outside the nest located between the nest entrance and the food baits. In order to reduce variability in foraging activity during a 24-h cycle, number of workers for active colonies was counted at four different sampling periods, twice in the night and twice in the day. The value taken for each colony was the maximum number of workers counted at these four different periods. Least-squares linear regression was used to test the relationship between average forager number per species per year and mean annual precipitation. Data were log_10_ transformed to meet the assumptions of normality and homogeneity of variances.

#### Specialized seed eaters

Species included in this group are seed harvesters, which belong exclusively to the genus *Messor* and forage mainly on plant seeds [42,49]. Workers of these species are considerably larger than the generalist species (S1 Table and S1 Fig), showing highly polymorphic body sizes with continuous morphological variation [40]. The foraging activity of these species was monitored together with that of the generalist species in each site at the same three randomly-chosen plots of 20 × 15 m in August 2007 and September 2008. The *Messor* species occurred more often at the seed baits compared to the species categorized as generalists (S3 Fig).

The foraging activity of the specialized seed eaters was also monitored independently from that of the generalist species. *Messor* workers are larger and their mature colonies form conspicuous mounds, whose density and foraging activity can be measured by direct observations. At each site, ant foraging activity was observed in a plot of 100 × 100 m. The observations were conducted in two consecutive years at the same plots during September 2008 and 2009.

Colony density was estimated as the number of active colonies in the 100 × 100 m plot per site and was evaluated by visually searching for active colonies during early morning (06:00–11:00) and evening (16:00–19:00). When nest entrances of the same species were found to be less than 1 m apart, they were counted as a single nest. For each year, least-squares linear regression was used to test the relationship between number of colonies per site and mean annual precipitation.

Forager number per colony was estimated by counting the number of workers for 20 randomly selected colonies per site at the 100 × 100 m plots. As for the generalist species, forager number was measured as the maximum number of workers found anywhere along the foraging trails and was counted at four different sampling periods during the day, twice in the morning and twice in the evening. The relationship between average forager number per species and annual precipitation was tested using linear regression for each year separately.

In order to examine the differences between the two functional groups in the matching between forager number and primary productivity, the slopes of the linear regressions between mean forager number per site and mean annual precipitation were compared between specialized seed eaters *vs*. generalist species for the two years separately using ANCOVA. A significant interaction between functional group as a fixed factor and precipitation as the covariate indicates significant differences between the slopes.

### Competition intensity

Variation in competitive interactions among the ants along the gradient was evaluated using the spatial partitioning of species at food baits as well as the spatial distribution of ant colonies. Both methods were previously used to evaluate competitive interactions in ants indirectly (e.g. [50,51]). However, the first technique estimates present competitive interactions occurring during foraging at bait stations, while the latter could be either a cause or consequence of competitive interactions and therefore could reflect past or present competition. For the specialized seed eaters, only the method of spatial distribution of colonies was used, because foraging activity was not examined using baits at the 100 × 100 m plots, but only by direct observation.

#### Spatial partitioning of species at baits

Co-occurrence patterns among species at baits were evaluated to determine whether the species aggregate or segregate in space. These patterns were analyzed using EcoSim, version 7 [52], which tests for non-random species co-occurrence patterns in a presence-absence matrix by randomizing the observed matrix (5000 times in our analyses). Only species that occurred in at least 5% of the baits per site and occupied simultaneously the same plots were included in the analysis. To quantify the tendency of species to not co-occur, the Stone and Roberts [53] C-score index was used, which measures the average number of checkerboard units (i.e., pairs of baits occupied by two different species) among the species. In a competitively structured community, the C-score should be significantly larger than expected by chance. The null model algorithm used in this analysis consisted of equiprobable columns (samples) and fixed row (species) sums, allowing the number of species in a sample to vary while keeping the same average number of species for the samples. This null model was suggested as the most appropriate algorithm for analyzing samples such as food baits [50]. In addition, Standardized Effect Size (SES) values were used to compare results among sites. Here, the observed matrix is rescaled as the number of standard deviation units above or below the mean of the simulated values. Assuming a normal distribution of deviations, approximately 95% of the SES values should fall between -1.96 and 1.96. Values larger than 1.96 indicate non-random segregation, and values lower than -1.96 indicate non-random aggregation of species. SES values may be confounded by forager number or colony density per site, which could limit the potential for competitive encounters among species without having to actively avoid competitors. Therefore, the correlation between SES and colony density or forager number for the two years was tested using Pearson’s correlation coefficient.

#### Spatial distribution of ant colonies

The distance between ant colonies and their nearest neighbors was measured at each site for the generalist species and the specialized seed eaters separately. The minimum observed distances between colonies of all species per plot were then compared to random minimum distances, which were generated using a program code written in MATLAB R2009b (The MathWorks Inc., Natick, MA, USA). The MATLAB code is available upon request. In order to compare the different values among sites, the standard variation (Z) of Nearest Neighbor Distance was calculated using the method of Clark and Evans [54]:

$$Z=(NND_{obs}–NND_{sim})/sdNND_{sim}$$

where *NND*_*obs*_ is the mean of observed minimum distances per plot, *NND*_*sim*_ is the mean of 5000 simulated minimum distances per plot and *sdNND*_*sim*_ is the standard deviation of these random distances. Z values between -1.96 and 1.96 indicate random distribution of colonies. Values larger than 1.96 indicate colony segregation (i.e., uniform distribution) and values below -1.96 indicate colony aggregation (i.e., clumped distribution). Mean values for each plot were then averaged for each site per year. The relationship between *NND* and annual precipitation or colony density was tested using linear regression.

All statistical analyses were conducted using JMP 8 (SAS Institute Inc., Cary, NC, USA) unless otherwise indicated.

## Results

### Ant colony density and forager number

**Fig 1:**
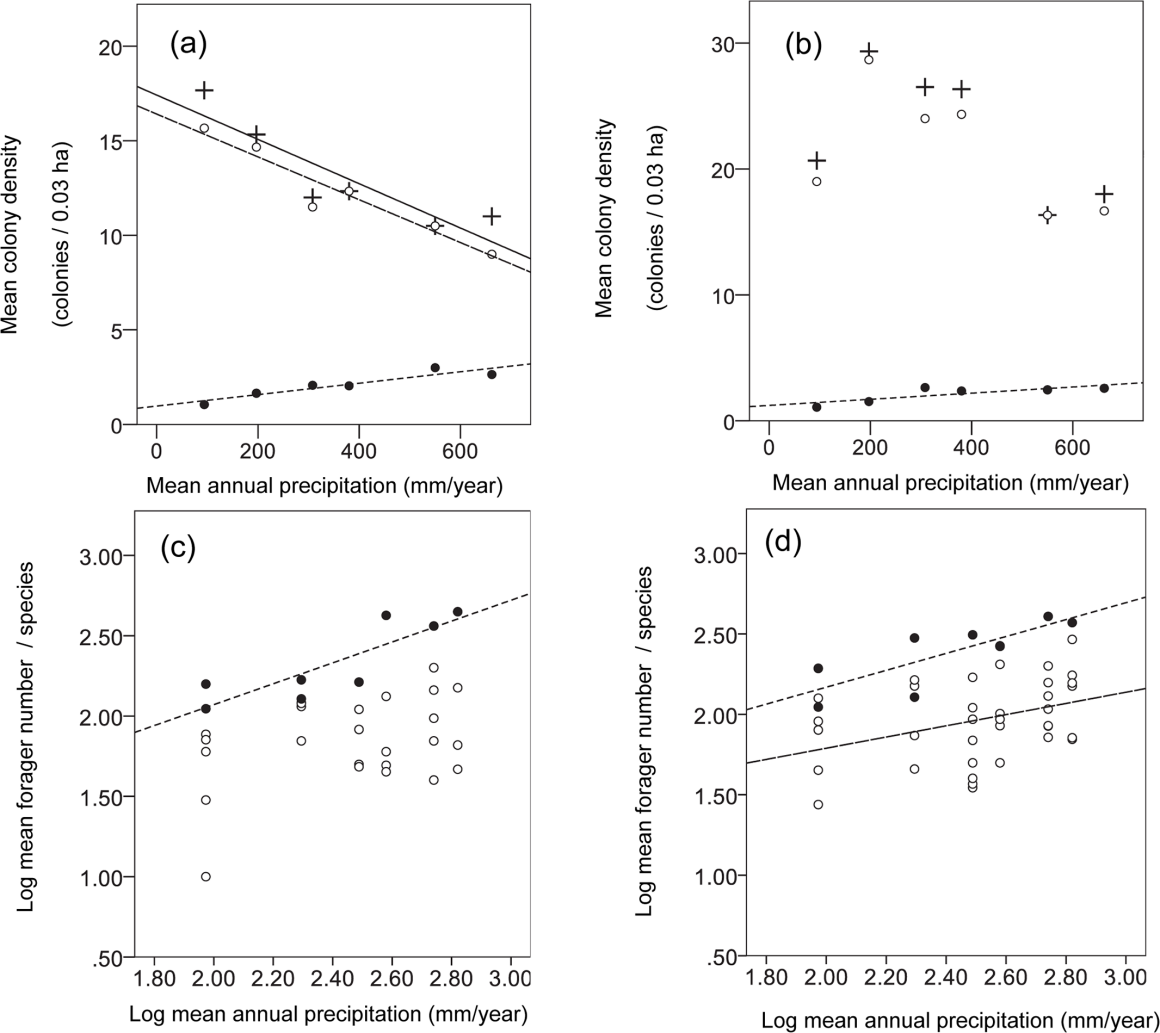
Relationships between *colony density* or *forager number per species* and mean annual precipitation in 2007 (a,c) and 2008 (b,d) of the generalist species (open circles, dashed line) or both functional groups when monitored together (plus sign, solid line) in the 20 × 15 m plots. The same relationships were also obtained for the specialized seed eaters (filled circles, dotted line) in the 100 × 100 m plots in 2008 (b,d) and 2009 (a,c) and standardized according to the 20 × 15 m plots.

A total of 19,850 individuals from 207 colonies, and 44,722 individuals from 387 colonies were recorded in 2007 and 2008, respectively. Seventeen ant species were found across the different sites, most of which (13 species) belong to the generalist category (S1 Table), and mean species richness per site was approximately 8 (range 7–9). Overall, mean colony density of the two functional groups for the two years decreased with increasing mean annual precipitation (linear regression: *y* = 22.78–0.013*x*, F_1,4_ = 8.87, r^2^ = 0.69, P = 0.041). When analyzed separately for each year, colony density decreased with mean annual precipitation in 2007 (linear regression: *y* = 17.42–0.012*x*, F_1,4_ = 16.52, r^2^ = 0.81, P = 0.015; Fig 1A) but not in 2008 (linear regression: F_1,4_ = 2.12, r^2^ = 0.35, P = 0.22; Fig 1B). In contrast, mean forager number for the two years increased with mean annual precipitation (linear regression: *y* = 72.13+0.138*x*, F_1,4_ = 17.40, r^2^ = 0.81, P = 0.014).

#### Generalist species

Similar to the patterns shown above for both functional groups, colony density decreased with mean annual precipitation in 2007 (linear regression: *y* = 16.41–0.011*x*, F_1,4_ = 46.26, r^2^ = 0.92, P < 0.005; Fig 1A). In 2008, the linear relationship was not significant (linear regression: F_1,4_ = 1.95, r^2^ = 0.33, P = 0.23; Fig 1B). Forager number per species increased with mean annual precipitation in 2008 (linear regression: *logy* = 2.52+0.348*logx*, F_1,35_ = 5.59, r^2^ = 0.14, P = 0.024; Fig 1D) but not in 2007 (linear regression: *logy* = 2.41+0.321*logx*, F_1,25_ = 3.52, r^2^ = 0.12, P = 0.072; Fig 1C).

#### Specialized seed eaters

Colony density increased with mean annual precipitation in 2008 (*y* = 0.97+0.003*x*, linear regression: F_1,4_ = 25.34, r^2^ = 0.86, P < 0.01; Fig 1B) and 2009 (*y* = 1.22+0.002*x*, linear regression: F_1,4_ = 7.33, r^2^ = 0.65, P = 0.053; Fig 1A). Similarly, forager number per species increased with mean annual precipitation in both years (linear regression: 2008, *logy* = 2.57+0.525*logx*, F_1,6_ = 10.88, r^2^ = 0.64, P = 0.016; Fig 1D; 2009, *logy* = 1.77+0.650*logx*, F_1,6_ = 16.28, r^2^ = 0.73, P < 0.001; Fig 1C).

As predicted, the slope of increase in mean forager number with precipitation was significantly greater for the specialized seed eaters than for the generalist species (2007: β_specialists_ = 0.594 ± 0.479, β_generalists_ = 0.053 ± 0.136; ANCOVA: functional group × mean annual precipitation, F_1,8_ = 9.07, P = 0.017; 2008: β_specialists_ = 0.415 ± 0.242, β_generalists_ = 0.117 ± 0.113; ANCOVA: functional group × mean annual precipitation, F_1,8_ = 9.57, P = 0.015) (Fig 1C and 1D).

### Competition intensity

#### Generalist species

**Fig 2:**
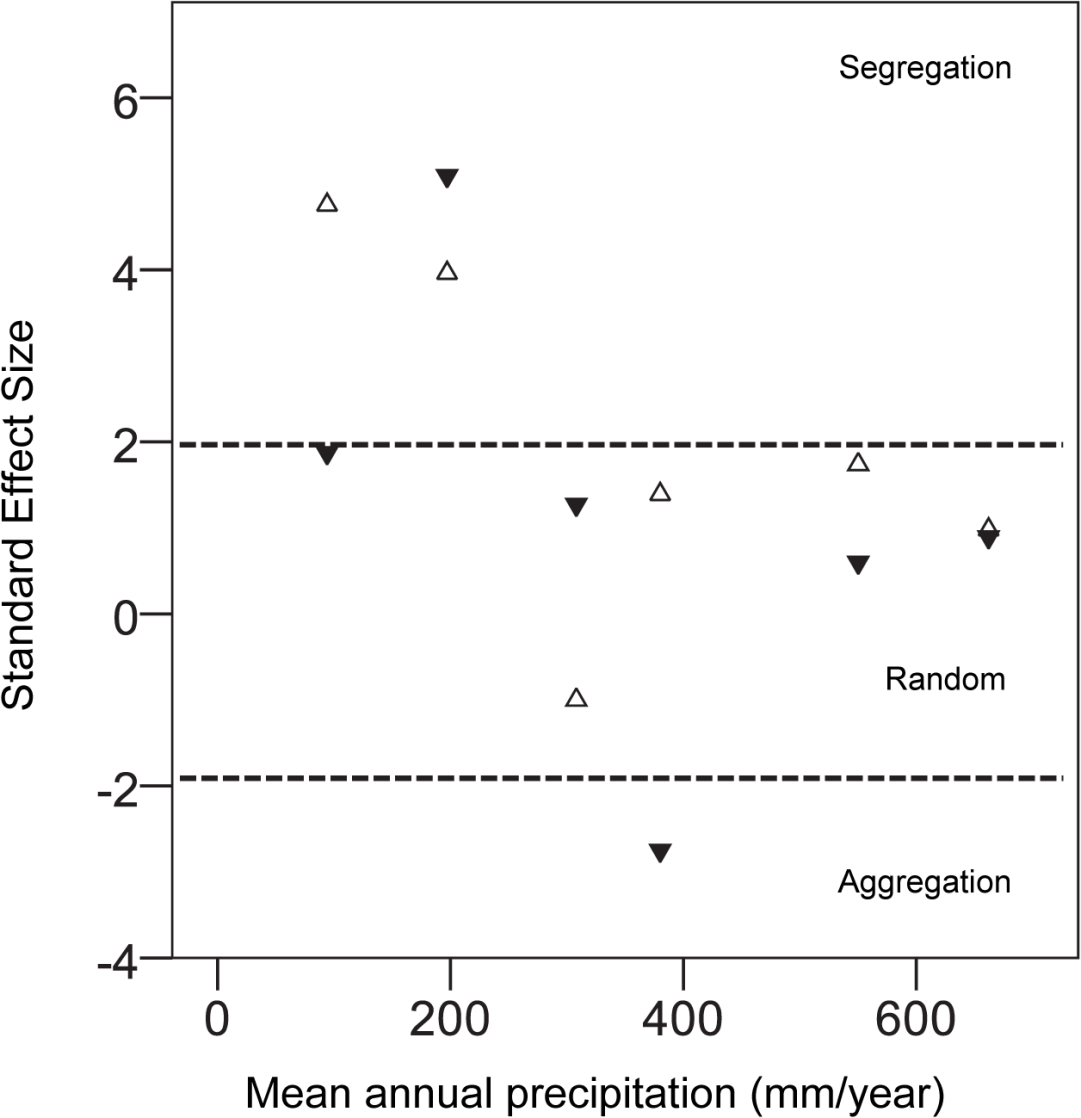
The relationship between Standardized Effect Size values of co-occurrence patterns between generalist species and mean annual precipitation in 2007 (open triangles) and 2008 (filled triangles). SES values between -1.96 and 1.96 indicate random species co-occurrence patterns in the experimental baits. Values above 1.96 and below -1.96 indicate non-random species segregation and aggregation, respectively.

The generalist species were found more often in the honey than in seed baits (S3 Fig). Species co-occurrence patterns were similar between the two years (S2 Table). The observed C-score values were significantly higher than the simulated values in the two most arid sites, Sede-Boqer and Hatzerim, indicating a tendency of species in these sites to segregate. In the more productive sites, however, the observed C-score values were not significantly different from random. A similar pattern was obtained using Standardized Effect Size (SES) values to compare among sites. SES values were larger than 1.96 only for the two arid sites, indicating non-random segregation of species (Fig 2). In the second year however, the value for the arid site (Sede-Boqer) was slightly lower (1.89). In the other sites, the distribution of species at baits was not significantly different from random. In addition, SES values for the two years did not correlate with colony density (Pearson’s r = 0.12, P = 0.72, n = 12) or forager number (Pearson’s r = -0.19, P = 0.55, n = 12).

**Fig 3:**
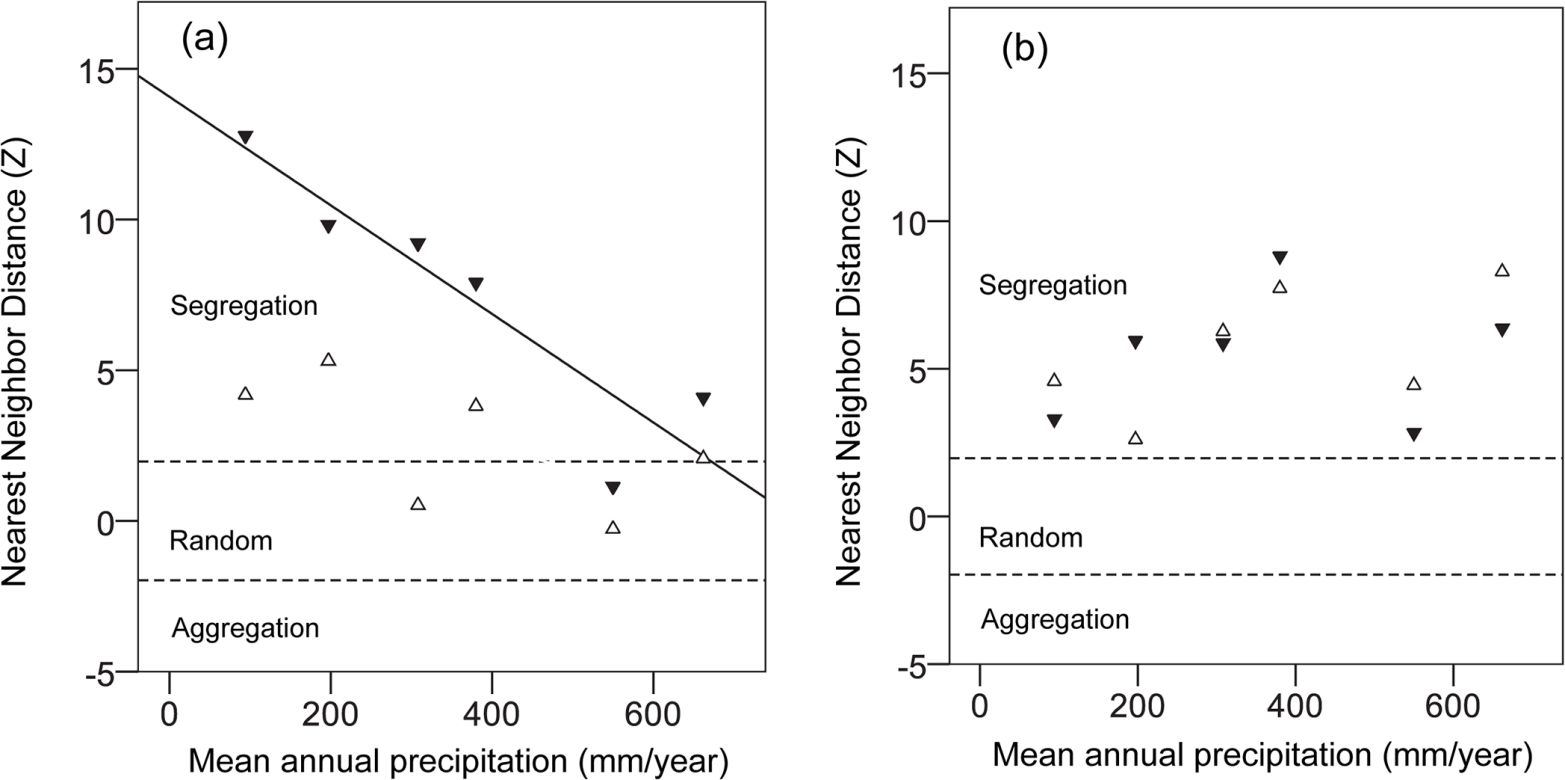
The relationship between the standard score (Z) of Nearest Neighbor Distance and mean annual precipitation for the generalist species (a) and specialized seed eaters (b) in 2007 (open triangles) and 2008 (filled triangles, solid line). Z values between -1.96 and 1.96 indicate random distribution of colonies. Values above 1.96 and below -1.96 indicate colony segregation and aggregation, respectively.

Minimum distance between colonies was not significantly affected by mean annual precipitation in 2007 (linear regression: F_1,4_ = 2.41, r^2^ = 0.376, P = 0.19), but the relationship was negative in 2008 (linear regression: F_1,4_ = 20.28, r^2^ = 0.84, P = 0.011) (Fig 3A). In addition, minimum distance between colonies for the two years correlated positively with colony density (Pearson’s r = 0.77, P = 0.003, n = 12), indicating increasing uniformity in nest distribution with increasing colony density.

#### Specialized seed eaters

In both 2008 and 2009, nest distribution was uniform and minimum distance between colonies was not affected by mean annual precipitation (linear regression: 2008, F_1,4_ = 0.074, r^2^ = 0.018, P = 0.80; 2009, F_1,4_ = 2.100, r^2^ = 0.34, P = 0.22; Fig 3B). In addition, minimum distance between colonies for the two years did not correlate with colony density (Pearson’s r = 0.36, P = 0.26, n = 12).

## Discussion

Abundance of organisms can vary in conflicting ways along gradients of resource availability. Along such gradients, increased resource supply is predicted to positively affect the relative abundances of species [4,6,55]. Our results revealed a different pattern by which colony density of ground-dwelling ant species decreased with productivity, which might be attributed to an observed increase in foraging activity, a size measurement of the forager caste of individual colonies. However, a separate analysis for generalist species *vs*. specialized seed eaters revealed that these distinct groups exhibit opposite patterns of response. These contrasting patterns provide support for two opposing hypotheses regarding the effect of resource availability on ant abundance.

### Specialized seed eaters and the ‘productivity hypothesis’

The increase in colony density of specialized seed eaters with increasing productivity is consistent with the prediction of the productivity hypothesis [4], namely, that the abundance of ant colonies is constrained by resource availability. These findings suggest that resource availability plays an important role in structuring the assemblage of specialized seed eaters in this arid region. Similar trends were obtained in a number of studies of ant communities, in which resource availability along geographical gradients was shown to limit colony density [4,56,57].

With the increase in colony density and foraging activity along the productivity gradient, it is possible that competition intensity will increase with increased resource availability, due to potentially more frequent encounters among the ant colonies or greater aggressiveness, which could be exhibited as a result of increased forager number (e.g. [51,58]). Alternatively, competition intensity could decrease with increased resource availability, due to a more intense 'struggle' for the limited resources under lower levels of resource availability. However, our results regarding nearest-neighbor distances between colonies of seed-harvesting ants indicate significant over-dispersion at all sites, without any effect of either precipitation or colony density. This suggests that competition is intense but does not differ among sites, despite differences in resource availability. As competition intensity in the specialized seed eaters was measured using colony nearest neighbor estimates, it is not clear whether the observed over-dispersion of colonies is maintained due to exploitative or interference competition, such as selective killing of new queens or elimination of smaller neighbor colonies. The nearest-neighbor distance measurements may reflect past or present competition, and we can only speculate about the mechanisms causing this over-dispersion pattern.

Results of another study conducted across the same sites [40], revealed that mean foraging distances of the seed-harvesting ant species decreases with an increase in precipitation, despite a higher foraging activity. This decrease in foraging distance could be explained either by an increase in food resources, which could allow for the shortening of foraging trails, or by an increase in competition intensity by the more dense and active colonies. This result together with the nearest-neighbor distance analyses suggest that competition intensity does not decrease along this productivity gradient.

The observed pattern of colony dispersion could be explained not only by competition intensity, but also by the patchy distribution of food resources. However, this in unlikely in our system for the following reasons: First, if indeed the harvester ant colonies were concentrated in rich food patches, then the observed dispersion should have been clumped instead of uniform. Secondly, personal observations at the study sites indicate that rich seed reserves are more patchily distributed at the low-productivity region. This suggests that if harvester ant colonies were indeed concentrated in rich food patches, then the correlation between NND and precipitation would have been negative and the foraging distances of the *Messor* spp. would have been similar along the productivity gradient, which was not the case.

### Generalist ants and the ‘productivity-based thinning hypothesis’

The generalist species revealed a contrasting pattern to that of the specialized seed eaters: while mean forager number per colony increased with increasing productivity, colony density decreased. These results are consistent with the predictions of the productivity-based thinning hypothesis, according to which foraging activity per colony increases and colony density decreases with increasing resource availability. Furthermore, according to this hypothesis, competition intensity between colonies may remain similar with increased resource availability due to a decrease in colony density and a concurrent increase in foraging activity. Our results of the spatial partitioning of species at baits and spatial distribution of ant colonies suggest, however, a decrease in the intensity of competitive interactions along the productivity gradient. This is indicated first by the fact that the generalist species tended to co-occur less frequently than expected by chance in baits at the low-productivity sites, suggesting that in these sites they tended to avoid inter-specific encounters. Second, the nearest-neighbor distances were positively correlated with colony density in the two years, but negatively correlated with productivity, indicating that colony dispersion of the generalist species changed from an over-dispersed to a more randomly-dispersed pattern with increased productivity.

Although competition may be an important factor explaining the decrease in colony density, we also should consider the distinction between competition intensity and its importance at the community level. As suggested by Welden and Slauson [59], competition intensity is the sum or average of the amount of strain experienced by individuals as a result of resource use by other individuals. Competition importance, however, is the effect of competition on the distribution and spatial patterning of individuals and species along environmental gradients *relative to the effect of other processes* (e.g. disturbance, predation). We propose that the decrease in colony density along this resource gradient could be the result of an increase in the importance rather than intensity of competition, past or present, among the more active colonies of the generalist species. More experiments are needed in order to further support or refute the productivity-based thinning hypothesis, for instance by experimentally manipulating foraging activity or augmenting food resources at the different sites along the productivity gradient.

Another possible explanation to the observed decrease in colony density of the generalist species with increased productivity could be related to changes in the abundance of the specialized seed eaters in the ant assemblages. This decrease might be accounted for by interspecific competition with these species, with which they share resources and which exhibit an increase in both colony density and foraging activity along the productivity gradient. The specialized seed eaters, which use seeds as a greater fraction of their diet, are larger than the generalist species and have highly polymorphic worker sizes. Thus, they might be more efficient foragers, as they can carry a greater diversity of food particles to greater distances [27,31,40,42]. In addition, as suggested in other studies, small numbers of larger-sized species with larger colonies could dominate energy use in eusocial insect communities [31,60]. Although larger workers are more costly to produce [61], they may have a longer lifespan [60], which could decrease worker turnover and increase colony size (i.e. total number of workers and alates per colony) [31,60,62]. Interestingly, examination of the relationship between two measured size components of the forager caste, forager body size and forager number per colony, revealed a positive linear relationship, in which forager number increases with species’ body size (S4 Fig), a similar relationship to that found previously between colony size and worker body size [31,60,62,63].

Finally, the decrease in colony density of the generalist species with increased productivity could be a sampling artifact. For example, the ability to detect ant colonies around the food baits may decrease with an increase in productivity due to greater plant height and habitat complexity, or due to changes in the response of the ant species to the experimental baits. In that case, observed foraging distances to the experimental baits should decrease with an increase in site productivity. However, our measurements of generalist species revealed that there was no significant negative correlation between mean annual precipitation and foraging distance (U. Segev, unpublished data), suggesting that sampling artifacts are unlikely to have caused the pattern observed in our study.

### Conclusions

Our study demonstrates that colony density of ground-dwelling ants is affected by resource availability, but that the nature of the effect differs in specialized seed eaters and generalist species. The contrasting responses found in these two groups may be attributed to greater resource use efficiency of the specialized seed eaters, which enables better matching between resource availability and its conversion into workers, compared to the generalist species. These findings stress the importance of considering the role of functional groups in studies of community structure. Our findings also indicate a directional change in competition intensity along the productivity gradient in one of the functional groups- it decreased with increasing productivity for the generalists, but was uncorrelated with productivity for the specialized seed eaters. Further studies measuring interference and exploitative competition experimentally at the community level and across a geographical scale are needed to unravel the variation in competition within a taxocene along latitudinal gradients [64,65]. We studied patterns along a short geographic gradient with a similar ant species pool, in which annual rainfall and plant biomass are highly correlated and change dramatically within a very short distance. This approach minimizes potential confounding effects due to climatic effects, habitat heterogeneity, different regional species pools and biogeographic history. Consistency in our results over the two sampling years supports the validity of the observed patterns. Finally, unraveling causes of change in species abundances in different assemblages is among the most general and important endeavors in community ecology and macroecology [2]. Here, we propose that energy-based variables and biotic interactions are both important in explaining these patterns of change. Such a mechanistic approach is necessary for understanding large-scale ecological patterns.

## Supporting Information

S1 Fig Relationships between forager body size and mean annual precipitation for generalist species and specialized seed-eaters. (DOCX)

S2 Fig Relationships between plant biomass and precipitation (Figure a) or between ambient temperature and precipitation (Figure b). (DOCX)

S3 Fig Proportion of total occurrences of ant species at the different sites according to bait type. (DOCX)

S4 Fig The allometric scaling relationship between forager number and forager body size. (DOCX)

S1 Table Averages of colony density, forager number (±se) and forager size (mm) (±se) of the generalist species monitored in the study sites in 2007–2008 and the specialized seed-eaters in 2008–2009. (DOCX)

S2 Table Co-occurrence patterns of generalist ant species at food baits in the years 2007 and 2008. (DOCX)

S3 Table Species activity monitored at the experimental baits for the generalist species (2007–2008) and at natural food patches for the specialized seed-eaters (2008–2009). (DOCX)
