# Supplementary material for: Ant Abundance along a Productivity Gradient: Addressing Two Conflicting Hypotheses
Source: PLoS One. 2015 Jul 15;10(7):e0131314. doi: 10.1371/journal.pone.0131314 (PMC4503676; doi:10.1371/journal.pone.0131314)
Supplement: S1 Fig — (DOCX) [file pone.0131314.s001.docx]

**Figure S1.** Relationships between forager body size and mean annual precipitation for generalist species (filled circles) and specialized seed-eaters (open circles). Values represent the two-year average for all species at the different sites (±SE). See S1 Table for more information on the way body size was measured.

Mean annual precipitation (mm/year)

Log mean forager size
