# Supplementary material for: Ant Abundance along a Productivity Gradient: Addressing Two Conflicting Hypotheses
Source: PLoS One. 2015 Jul 15;10(7):e0131314. doi: 10.1371/journal.pone.0131314 (PMC4503676; doi:10.1371/journal.pone.0131314)
Supplement: S2 Fig — (DOCX) [file pone.0131314.s002.docx]

**S2 Figure.** The relationship between precipitation, plant biomass and ambient temperature (see Table 1 for more details on data collection). (a) Mean plant biomass positively increases with mean annual precipitation (Linear regression: *y*=36.91+0.456*x*, r^2^ = 0.92, F_1,4_ = 44.52, *P* < 0.005). (b) Mean maximum temperatures and mean annual precipitation, however, are not correlated (Linear regression: *y*=25.35+0.002*x*, r^2^ = 0.051, F_1,4_ = 0.21, *P* = 0.67).

Plant biomass (g/m^2^/y)

(a)

Mean annual precipitation (mm/year)

(b)

Mean annual precipitation (mm/year)

Mean maximum temperature (°C)
