# Supplementary material for: Ant Abundance along a Productivity Gradient: Addressing Two Conflicting Hypotheses
Source: PLoS One. 2015 Jul 15;10(7):e0131314. doi: 10.1371/journal.pone.0131314 (PMC4503676; doi:10.1371/journal.pone.0131314)
Supplement: S3 Fig — (DOCX) [file pone.0131314.s003.docx]

**S3 Figure.** Proportion of total occurrences of ant species at the different sites according to bait type. The specialized seed-eaters (a) occurred more often in seed baits than the generalist species (b) (Mann-Whitney U test: *P* < 0.005). Data summed over three surveys: Summer 2007, Spring 2008 and Summer 2008.

Proportion of occurrences in baits

*a. Specialized seed-eaters*

*b. Generalist species*

Species
