# Supplementary material for: Ant Abundance along a Productivity Gradient: Addressing Two Conflicting Hypotheses
Source: PLoS One. 2015 Jul 15;10(7):e0131314. doi: 10.1371/journal.pone.0131314 (PMC4503676; doi:10.1371/journal.pone.0131314)
Supplement: S4 Fig — (DOCX) [file pone.0131314.s004.docx]

**S4 Figure.** The allometric scaling relationship between forager number and forager body size. Values represent the two-year average for all species. Linear regression: *y*=1.553+0.817*x*, r^2^ = 0.48, F_1,15_ = 13.96, *P* < 0.005.

Log forager number

Log forager size
