# Supplementary material for: Ant Abundance along a Productivity Gradient: Addressing Two Conflicting Hypotheses
Source: PLoS One. 2015 Jul 15;10(7):e0131314. doi: 10.1371/journal.pone.0131314 (PMC4503676; doi:10.1371/journal.pone.0131314)
Supplement: S1 Table — (DOCX) [file pone.0131314.s005.docx]

**S1 Table.** Averages of colony density^§^, forager number^#^ (±se) and forager size^&^ (mm) (±se) of the generalist species monitored in the study sites in 2007-2008 and the specialized seed-eaters in 2008-2009.

| **Species** | **Trophic**^*^ | **Variable** | **Sede Boqer** | **Hatzerim** | **Lahav** | **Amatzia** | **Karei Deshe** | **Ramot Menashe** |
| --- | --- | --- | --- | --- | --- | --- | --- | --- |
| **Specialized seed-eaters** | | | | | | | | |
| *Messor arenarius*^‡^ | SH | Colony density | 4.5 | 6.5 |  |  |  |  |
|  |  | Forager number | 111±16 | 128±16 |  |  |  |  |
|  |  | Forager size | 11.8±0.17 | 12.16±0.16 |  |  |  |  |
| *Messor dentatus* | SH | Colony density |  |  |  |  | 90 |  |
|  |  | Forager number |  |  |  |  | 381±16 |  |
|  |  | Forager size |  |  |  |  | 7.75±0.28 |  |
| *Messor ebeninus* | SH | Colony density | 31 | 46.5 |  |  |  |  |
|  |  | Forager number | 172±26 | 255±38 |  |  |  |  |
|  |  | Forager size | 7.18±0.15 | 7.57±0.13 |  |  |  |  |
| *Messor semirufus* | SH | Colony density |  |  | 78.5 | 73.5 |  | 87 |
|  |  | Forager number |  |  | 259±24 | 341±28 |  | 399±15 |
|  |  | Forager size |  |  | 7.47±0.16 | 7.47±0.14 |  | 7.82±0.15 |
| **Generalist species** | | | | | | | | |
| *Lepisiota syriaca* | GF | Colony density |  |  | 1 | 1.5 | 1.5 | 10.5 |
|  |  | Forager number |  |  | 50 | 93±7 | 147±61 | 72±7 |
|  |  | Forager size |  |  | 3.72±0.09 | 3.65±0.07 | 3.20±0.07 | 3.76±0.05 |
| *Cardiocondyla batesii* | Opp. | Colony density | 1 |  |  |  |  |  |
|  |  | Forager number | 45±15 |  |  |  |  |  |
|  |  | Forager size | 2.37±0.07 |  |  |  |  |  |
| *Crematogaster jehovae* | GF | Colony density |  |  |  | 13 | 12 | 3 |
|  |  | Forager number |  |  |  | 127±24 | 91±9 | 66±12 |
|  |  | Forager size |  |  |  | 3.82±0.06 | 3.92±0.04 | 3.73±0.08 |

**S1 Table cont.**

| **Species** | **Trophic**^*^ | **Variable** | **Sede Boqer** | **Hatzerim** | **Lahav** | **Amatzia** | **Karei Deshe** | **Ramot Menashe** |
| --- | --- | --- | --- | --- | --- | --- | --- | --- |
| *Temnothorax arenarius* | GF | Colony density | 2.5 |  | 1 |  |  |  |
|  |  | Forager number | 28±6 |  | 40 |  |  |  |
|  |  | Forager size | 2.79±0.1 |  | 2.96±0.12 |  |  |  |
| *Monomorium sommieri* | GF | Colony density | 13.5 | 7.5 |  |  |  |  |
|  |  | Forager number | 77±9 | 97±10 |  |  |  |  |
|  |  | Forager size | 2.92±0.05 | 2.49±0.02 |  |  |  |  |
| *Monomorium salomonis* | GF | Colony density | 23 |  | 16 | 19.5 | 3 | 8.5 |
|  |  | Forager number | 99±12 |  | 83±23 | 74±12 | 85±24 | 65±8 |
|  |  | Forager size | 3.33±0.02 |  | 2.84±0.03 | 3.23±0.03 | 3.08±0.03 | 2.77±0.04 |
| *Monomorium advena* | GF | Colony density |  |  | 1.5 |  |  |  |
|  |  | Forager number |  |  | 40±6 |  |  |  |
|  |  | Forager size |  |  | 1.95±0.04 |  |  |  |
| *Monomorium clavicorne* | GF | Colony density |  |  |  |  | 0.5 |  |
|  |  | Forager number |  |  |  |  | 70 |  |
|  |  | Forager size |  |  |  |  | 1.26±0.01 |  |
| *Monomorium dentigerum* | GF | Colony density |  | 12 | 1.5 | 11 | 2.5 | 4.5 |
|  |  | Forager number |  | 118±12 | 110±27 | 198±26 | 166±28 | 163±22 |
|  |  | Forager size |  | 3.70±0.13 | 3.63±0.09 | 3.47±0.08 | 3.38±0.10 | 3.55±0.10 |
| *Pheidole pallidula* | O^†^ | Colony density |  | 41 | 2 |  | 0.5 | 2.5 |
|  |  | Forager number |  | 142±9 | 170±36 |  | 130 | 293±34 |
|  |  | Forager size |  | 2.86±0.01 | 3.02±0.03 |  | 2.55±0.02 | 2.60±0.02 |
| *Plagiolepis sp.* | GF | Colony density |  |  | 10.5 | 1.5 | 5 |  |
|  |  | Forager number |  |  | 41±4 | 50±35 | 72±14 |  |
|  |  | Forager size |  |  | 1.90±0.04 | 1.88±0.03 | 1.94±0.02 |  |

| **Species** | **Trophic**^*^ | **Variable** | **Sede Boqer** | **Hatzerim** | **Lahav** | **Amatzia** | **Karei Deshe** | **Ramot Menashe** |
| --- | --- | --- | --- | --- | --- | --- | --- | --- |
| *Tapinoma sp.* | GF | Colony density | 0.5 | 0.5 |  | 1.5 |  | 0.5 |
|  |  | Forager number | 10 | 70 |  | 77±38 |  | 150 |
|  |  | Forager size | 2.50±0 | 3.63±0.18 |  | 3.44±0.08 |  | 3.49±0.10 |
| *Tetramorium sp.* | Opp. / GF | Colony density | 10.5 | 4 | 6 | 6 | 9.5 | 1.5 |
|  |  | Forager number | 75±11 | 46±6 | 88±17 | 91±22 | 118±23 | 157±52 |
|  |  | Forager size | 2.66±0.03 | 2.99±0.05 | 2.69±0.02 | 2.72±0.03 | 3.08±0.02 | 3.08±0.04 |

**S1 Table cont.**

§ Colony density was estimated as the total number of colonies per site averaged for the two years 2007-2008 (generalists; 20 × 15 m plots) or 2008-2009 (specialized seed eaters; 100 × 100 m plot).

# Forager number was estimated as the averages for all colonies sampled in the two years 2007-2008 (generalists) or 2008-2009 (specialized seed eaters).

& forager body size was assessed by measuring average body length, from the apex of the head to the end of the gaster of 5-30 randomly selected pin-mounted individuals from one to seven colonies per species per site, using a stereo microscope equipped with an ocular micrometer (0.01 mm scale). For dimorphic species, only minor workers were measured. Major workers were excluded as they were found in only one species (*Pheidole pallidula*) and in small numbers.

* Trophic levels of the ant species, classified at the genus level for the dominant trophic habit (based on Brown 2000): SH = Seed harvesters, GF = Generalized foragers, Opp. = Opportunists, O = Omnivores.

† *Pheidole pallidula* could be considered as either omnivorous or seed harvesting species. We decided to classify this species as omnivorous, based on feeding observations in this study and in other studies conducted in this region (Segev 2010; Segev and Ziv 2012).

‡ Measurements of worker number for this species were underestimated due to its mixed foraging strategy of both solitary and group foraging and the difficulty in following individual workers in the field. Therefore, the values presented are the maximum worker number in six colonies monitored during the years 2007-2009.
