# Supplementary material for: Ant Abundance along a Productivity Gradient: Addressing Two Conflicting Hypotheses
Source: PLoS One. 2015 Jul 15;10(7):e0131314. doi: 10.1371/journal.pone.0131314 (PMC4503676; doi:10.1371/journal.pone.0131314)
Supplement: S2 Table — (DOCX) [file pone.0131314.s006.docx]

**S2 Table.** Co-occurrence patterns of generalist ant species at food baits in the years 2007 and 2008. In a competitively structured community, C-score values should be significantly higher than expected by chance. Means of simulated C-scores were produced following 5000 randomizations of the observed presence-absence matrix. *P* values are for C_obs_ > C_sim_. See Table S3 for more information on species activity at the experimental baits.

|  | **2007** | | | **2008** | | |
| --- | --- | --- | --- | --- | --- | --- |
| **Site (mm)** | **Observed**  **C-score** | **Mean of**  **simulated C-score** | ***P* value** | **Observed**  **C-score** | **Mean of**  **simulated C-score** | ***P* value** |
| 94 | 0.505 | 0.233 | < 0.001 | 0.438 | 0.365 | 0.044^§^ |
| 197 | 0.503 | 0.327 | < 0.001 | 0.189 | 0.078 | < 0.005 |
| 308 | 0.237 | 0.292 | 0.89 | 0.662 | 0.648 | 0.1 |
| 380 | 0.573 | 0.535 | 0.058 | 0.765 | 0.841 | 0.997 |
| 550 | 0.361 | 0.265 | 0.071 | 0.692 | 0.682 | 0.163 |
| 662 | 0.667 | 0.645 | 0.402 | 0.513 | 0.453 | 0.474 |

§ In this site, when patterns of foraging activity in baits were compared, observed C-score values were found to be significantly higher than the simulated values at day time, but not significantly different from random at night time.
