# Supplementary material for: Ant Abundance along a Productivity Gradient: Addressing Two Conflicting Hypotheses
Source: PLoS One. 2015 Jul 15;10(7):e0131314. doi: 10.1371/journal.pone.0131314 (PMC4503676; doi:10.1371/journal.pone.0131314)
Supplement: S3 Table — (DOCX) [file pone.0131314.s007.docx]

**S3 Table.** Species activity monitored at the experimental baits for the generalist species (2007-2008) and at natural food patches for the specialized seed eaters (2008-2009). Measurements include the proportion of occurrences at baits^§^, Thermal Activity Breadth^#^ (TAB) and maximum number of foragers. Bold values represent species that were included in the co-occurrence patterns analyses.

| **Species** | **Trophic** | **Variable** | **Sede Boqer** | **Hatzerim** | **Lahav** | **Amatzia** | **Karei Deshe** | **Ramot Menashe** |
| --- | --- | --- | --- | --- | --- | --- | --- | --- |
| **Specialized seed-eaters**^*^ | | | | | | | | |
| *Messor arenarius* | SH | Occurrences | 0.05 | - |  |  |  |  |
|  |  | TAB | 20-30 | 22-35 |  |  |  |  |
|  |  | Max. foragers | 120 | 150 |  |  |  |  |
| *Messor dentatus* | SH | Occurrences |  |  |  |  | - |  |
|  |  | TAB |  |  |  |  | 20-33 |  |
|  |  | Max. foragers |  |  |  |  | 400 |  |
| *Messor ebeninus* | SH | Occurrences | 0.1 | 0.05 |  |  |  |  |
|  |  | TAB | 20-30 | 18-32 |  |  |  |  |
|  |  | Max. foragers | 300 | 450 |  |  |  |  |
| *Messor semirufus* | SH | Occurrences |  |  | 0.15 | 0.05 |  | 0.05 |
|  |  | TAB |  |  | 18-35 | 19-33 |  | 20-35 |
|  |  | Max. foragers |  |  | 550 | 500 |  | 500 |
| **Generalist species** | | | | | | | | |
| *Lepisiota syriaca* | GF | Occurrences |  |  | 0.05 | 0.1 | 0.075 | **0.25** |
|  |  | TAB |  |  | 22-31 | 30-32 | 25-31 | 25-35 |
|  |  | Max. foragers |  |  | 30 | 40 | 100 | 80 |
| *Cardiocondyla batesii* | Opp. | Occurrences | 0.1 |  |  |  |  |  |
|  |  | TAB | 26.5 |  |  |  |  |  |
|  |  | Max. foragers | 20 |  |  |  |  |  |
| *Crematogaster jehovae* | GF | Occurrences |  |  |  | **0.16** | **0.2** | **0.125** |
|  |  | TAB |  |  |  | 18-31 | 21-31 | 20-30 |
|  |  | Max. foragers |  |  |  | 350 | 200 | 60 |

**S3 Table cont.**

| **Species** | **Trophic** | **Variable** | **Sede Boqer** | **Hatzerim** | **Lahav** | **Amatzia** | **Karei Deshe** | **Ramot Menashe** |
| --- | --- | --- | --- | --- | --- | --- | --- | --- |
| *Temnothorax arenarius* | GF | Occurrences | 0.065 |  | 0.05 |  |  |  |
|  |  | TAB | 21-22.5 |  | 19 |  |  |  |
|  |  | Max. foragers | 25 |  | 2 |  |  |  |
| *Monomorium sommieri* | GF | Occurrences | **0.25** | **0.13** |  |  |  |  |
|  |  | TAB | 20-28 | 18-33 |  |  |  |  |
|  |  | Max. foragers | 150 | 70 |  |  |  |  |
| *Monomorium salomonis* | GF | Occurrences | **0.35** |  | **0.31** | **0.30** | 0.09 | **0.13** |
|  |  | TAB | 20-28 |  | 16-28 | 18-28 | 21-26 | 16-31 |
|  |  | Max. foragers | 250 |  | 70 | 250 | 100 | 80 |
| *Monomorium advena* | GF | Occurrences |  |  | 0.067 |  |  |  |
|  |  | TAB |  |  | 15-18 |  |  |  |
|  |  | Max. foragers |  |  | 20 |  |  |  |
| *Monomorium clavicorne* | GF | Occurrences |  |  |  |  | 0.1 |  |
|  |  | TAB |  |  |  |  | 26-30 |  |
|  |  | Max. foragers |  |  |  |  | 40 |  |
| *Monomorium dentigerum* | GF | Occurrences |  | **0.29** | 0.075 | **0.12** | 0.06 | **0.1** |
|  |  | TAB |  | 22-33 | 17-29 | 18-28 | 21-27 | 18-31 |
|  |  | Max. foragers |  | 100 | 120 | 350 | 200 | 100 |
| *Pheidole pallidula* | O^†^ | Occurrences |  | **0.53** | 0.175 |  | 0.05 | 0.1 |
|  |  | TAB |  | 15-26 | 15-24 |  | 23.5 | 17-31 |
|  |  | Max. foragers |  | 250 | 170 |  | 60 | 300 |
| *Plagiolepis sp.* | GF | Occurrences |  |  | **0.156** | 0.063 | **0.12** |  |
|  |  | TAB |  |  | 14-24 | 18-25 | 21-26 |  |
|  |  | Max. foragers |  |  | 50 | 60 | 100 |  |

**S3 Table cont.**

| **Species** | **Trophic** | **Variable** | **Sede Boqer** | **Hatzerim** | **Lahav** | **Amatzia** | **Karei Deshe** | **Ramot Menashe** |
| --- | --- | --- | --- | --- | --- | --- | --- | --- |
| *Tapinoma sp.* | GF | Occurrences | 0.1 | 0.05 |  | 0.05 |  | 0.05 |
|  |  | TAB | 20-24 | 27.5 |  | 23-26 |  | 20-25 |
|  |  | Max. foragers | 5 | 80 |  | 100 |  | 120 |
| *Tetramorium sp.* | Opp. / GF | Occurrences | **0.167** | 0.12 | **0.09** | **0.167** | **0.1** | 0.1 |
|  |  | TAB | 19–25 | 19-24 | 14-25 | 18-28 | 21-27 | 17-30 |
|  |  | Max. foragers | 90 | 20 | 150 | 250 | 250 | 150 |

§ Proportion of occurrences at experimental food baits was calculated using only plots in which species were active.

# Thermal Activity Breadth§ (TAB) was measured as ground temperature ranges (°C) at which 20% or more of the maximum number of foragers are active. Ground temperature was measured during sampling using an Enviro-Safe^®^ pocket thermometer (H-B Instrument Company, USA).

* Measurements for the specialized seed-eaters were obtained using a different data set in which we examined variability in foraging performance at the same study sites during the years 2007-2009 (Segev et al. 2014). For this functional group observations were made at natural food patches instead of experimental food baits.
